# Editorial: Community series in overeating and decision making vulnerabilities

**DOI:** 10.3389/fpsyg.2022.1101004

**Published:** 2023-01-20

**Authors:** Wei Li, Qinghua He

**Affiliations:** ^1^Ministry of Education (MOE) Key Laboratory of Cognition and Personality, Faculty of Psychology, Southwest University, Chongqing, China; ^2^Southwest University Branch, Collaborative Innovation Center of Assessment Toward Basic Education Quality at Beijing Normal University, Chongqing, China

**Keywords:** eating, decision making, obesity, overweight, fMRI

This Research Topic is the second volume of the “*Overeating and decision making vulnerabilities*” (He et al., 2019) (http://www.frontiersin.org/research-topics/4946/). We aimed to organize this Research Topic to gather new findings from various perspectives to deal with overeating and decision making vulnerabilities.

The prevalence of overweight and obesity has been growing globally. According to WHO, the adult population with obesity had exceeded those with normal weight in the world (World Health Organization, 2020). The COVID-19 and associated lockdown changed the way we live and communicate (Xiao et al., 2022), which may also promote obesity and associated metabolic diseases (Clemmensen et al., 2020). Anyhow, overweight and obesity was one of the top five factors affecting the disease risk (Kelley and Berridge, 2002; Rolls, 2007; Trinko et al., 2007; Volkow et al., 2008; Chen et al., 2018), which can impose a heavy burden the health system (He et al., 2014a,b, 2015; Gao et al., 2020; Liu et al., 2022).

In this series, we have collected five original articles (Duszka et al.; Guerrini Usubini et al.; Han et al.; Isham et al.; Kardas et al.) dealing with different aspects of food choice and obesity in community, such as food preference (Kardas et al.), portion estimation and choice (Duszka et al.), time perception and food comsumption (Isham et al.), cognitive inflexibility (Guerrini Usubini et al.), as well as brain and impulsivity (Han et al.).

Kardas et al. investigated the preferences and consumption of yogurt in terms of sensitivity to recognize sweetness and obesity in 160 children aged 7–9. They didn't find significant correlation between body weight and the frequency of fermented milk product consumption. However, they found that children who were normal- or under-weight was better recognize sweetness, and they preferred more plain unsweetened yogurt. They also call future studies to consider the influence of other food types and sensory attributes to shape food preferences in children (Kardas et al.).

Isham et al. surveyed 137 volunteers about their eating behavior and time perception (i.e., time estimation and passage of time judgement) during COVID-19 lockdown. Their results found that participants who judged time to pass slowly would tend to snack and overeat during lockdown. They discussed their findings though implications for eating disorders and potential time-based behavioral intervention strategies (Isham et al.).

Duszka et al. used two studies (*N* = 725 and *N* = 436, respectively) to understand the impacting factors of portion size preferences. In the first study, participants were asked to estimate the weight of the pictured foods, in which they found modest impacts of gender, BMI, age, and hunger on the estimation. In the second study, they asked the participants to do a portion choice task, and they found that males generally choose larger ones. Gender, external- and restrictive-eating behavior, age, hunger, but not BMI could also influence the portion size selection (Duszka et al.).

Guerrini Usubini et al. investigated and confirmed the mediation role of cognitive (in)flexibility in the relationship between anxiety and depression and emotional eating in 123 individuals with obesity. They reported the indirect mediated effect accounted for 19 and 12% of the variance in different models. Their study may help to guide new behavioral intervention method designed for obesity treatment (Guerrini Usubini et al.).

Han et al. investigated the impulsivity and resting state fMRI in 35 individuals with obesity and 31 controls. Direct comparison showed that individuals with obesity had a lower fractional amplitude of low-frequency fluctuations (fALFF) in the bilateral dorsolateral prefrontal cortex (dlPFC) than controls. They also found that the right dlPFC completely mediated the relationship between non-planning impulsiveness and BMI. Their results may provide a target for brain-based stimulation treating obesity (Han et al.).

All five articles covered in this Research Topic are important to understand the mechanism of overeating and decision making vulnerabilities from different perspectives. We hope they could be translated into effective ways to treat overweight and obesity as well as to guide a better life style.

## Author contributions

WL wrote the first draft. QH made critical revision. WL and QH approved the final version of the manuscript.

## References

[B1] Chen R. Li D. P. Turel O. Sørensen T. A. Bechara A. Li Y.. (2018). Decision making deficits in relation to food cues influence obesity: a triadic neural model of problematic eating [hypothesis and theory]. Front. Psychiatry 9, 264. 10.3389/fpsyt.2018.00264 29962976 PMC6010920

[B2] Clemmensen C. Petersen M. B. Sorensen T. I. A. (2020). Will the COVID-19 pandemic worsen the obesity epidemic? Nat. Rev. Endocrinol. 16, 469–470. 10.1038/s41574-020-0387-z 32641837 PMC7342551

[B3] Gao M. Lv J. Yu C. Guo Y. Bian Z. Yang R.. (2020). Metabolically healthy obesity, transition to unhealthy metabolic status, and vascular disease in Chinese adults: a cohort study. PLoS Med. 17, e1003351. 10.1371/journal.pmed.1003351 33125374 PMC7598496

[B4] He Q. Chen C. Dong Q. Xue G. Chen C. Lu Z.. (2015). Gray and white matter structures in the midcingulate cortex region contribute to body mass index in chinese young adults. Brain Struct. Funct. 220, 319–329. 10.1007/s00429-013-0657-9 24146133 PMC3995892

[B5] He Q. Gao X. Li Y. Chen H. (2019). Editorial: overeating and decision making vulnerabilities. Front. Psychol. 10, 587. 10.3389/fpsyg.2019.00587 30967812 PMC6438915

[B6] He Q. Xiao L. Xue G. wong s. Ames S. L. Bechara A. (2014a). Altered dynamics between neural systems sub-serving decisions for unhealthy food [original research]. Front. Neurosci. 8, 350. 10.3389/fnins.2014.00350 25414630 PMC4220120

[B7] He Q. Xiao L. Xue G. Wong S. Ames S. L. Schembre S. M.. (2014b). Poor ability to resist tempting calorie rich food is linked to altered balance between neural systems involved in urge and self-control. Nutr. J. 13, 92. 10.1186/1475-2891-13-92 25228353 PMC4172871

[B8] Kelley A. E. Berridge K. C. (2002). The neuroscience of natural rewards: relevance to addictive drugs. J. Neurosc. 22, 3306–3311. 10.1523/JNEUROSCI.22-09-03306.2002 11978804 PMC6758373

[B9] Liu X. Turel O. Xiao Z. He J. He Q. (2022). Impulsivity and neural mechanisms that mediate preference for immediate food rewards in people with vs without excess weight. Appetite 169, 105798. 10.1016/j.appet.2021.105798 34774966

[B10] Rolls E. (2007). Understanding the mechanisms of food intake and obesity. Obes. Rev. 8, 67–72. 10.1111/j.1467-789X.2007.00321.x 17316305

[B11] Trinko R. Sears R. M. Guarnieri D. J. DiLeone R. J. (2007). Neural mechanisms underlying obesity and drug addiction. Physiol. Behav. 91, 499–505. 10.1016/j.physbeh.2007.01.001 17292426

[B12] Volkow N. D. Wang G. J. Fowler J. S. Telang F. (2008). Overlapping neuronal circuits in addiction and obesity: evidence of systems pathology. Philos. Trans. R. Soc. B Biol. Sci. 363, 3191–3200. 10.1098/rstb.2008.0107 18640912 PMC2607335

[B13] World Health Organization. (2020). Overweight and Obesity. Available online at: https://www.who.int/news-room/fact-sheets/detail/obesity-and-overweight

[B14] Xiao Z. Chen Z. Chen W. Gao W. He L. Wang Q.. (2022). maladaptive changes in delay discounting in males during the Covid-19 Pandemic: the predictive role of functional connectome. Cereb. Cortex. 10.1093/cercor/bhab505 35059700 PMC9383225

